# Shared decision‐making on a ‘life‐and‐care plan’ in long‐term care facilities: research protocol

**DOI:** 10.1002/nop2.42

**Published:** 2016-03-06

**Authors:** Elena Mariani, Yvonne Engels, Raymond Koopmans, Rabih Chattat, Myrra Vernooij‐Dassen

**Affiliations:** ^1^ Scientific Institute for Quality of Healthcare (IQ healthcare)Radboud University Medical CentreP.O. Box 91016500 HBNijmegenThe Netherlands; ^2^ Department of Anaesthesiology, Pain and Palliative MedicineRadboud University Medical CentreP.O. Box 91016500 HBNijmegenThe Netherlands; ^3^ Department of Primary and Community CareRadboud University Medical CentreP.O. Box 91016500 HBNijmegenThe Netherlands; ^4^ Department of Psychology*Alma Mater* StudiorumUniversity of BolognaViale Berti Pichat 540127BolognaItaly

**Keywords:** Care plans, dementia, long‐term care, nursing, nursing homes, shared decision‐making

## Abstract

**Aim:**

To determine whether the number of residents' preferences and needs together with the actions taken to satisfy them recorded into their ‘life‐and‐care plans’ will increase and the process of shared decision‐making will improve the residents' psychosocial well‐being.

**Background:**

Shared decision‐making is defined as a process where healthcare professionals and patients make decisions together, using the best available evidence.

The aims of the present study were to assess the feasibility and acceptability of an SDM framework for care planning in long‐term care facilities and its potential effectiveness on the proportion of dementia residents whose own preferences and needs and the related actions, are known, satisfied and documented in their ‘life‐and‐care plans’.

**Design:**

The current project is a feasibility trial and it was approved in November 2013.

**Methods:**

Research subjects are triads composed of the resident with dementia, a family caregiver and the professional usually taking care for the resident. Professional caregivers of two nursing homes, one located in Italy and one in the Netherlands, will receive a specific training in SDM principles and will guide the SDM interview in the triad. The primary outcome will be the proportion of residents whose preferences and needs, together with the related actions to meet them, are known, documented and satisfied in their ‘life‐and‐care plans’.

**Trial registration:**

NCT02118701.

## Introduction

Long‐term care (LTC) residents with dementia have complex needs and can have difficulties in articulating them, since the ability to express their wishes is impaired (Hancock *et al*. 2006). This does not mean that they do not have their own preferences, that they are completely unable to articulate preferences and feelings (Carpenter *et al*. 2007), or that they are unable to answer simple questions about needs and preferences (Whitlatch 2010). While the abilities to answer fact‐based questions deteriorate after the early stages of dementia, the abilities to answer preference questions remain more stable over time (Whitlatch 2010). Some studies have shown that it is possible to assess individuals with dementia's personal preferences and to enhance their decision‐making involvement (Whitlatch 2010). Such studies have shown a positive link between the involvement of people suffering from dementia in decision‐making and their quality of life (Fetherstonhaugh *et al*. 2013).

## Background

Shared decision‐making (SDM) is defined as a process where the healthcare professional and the patient make decisions together, using the best available evidence (Charles *et al*. 1997, 1999, Elwyn *et al*. 2010). It requires sharing of information and agreement by both parties on the decisions taken (Elwyn *et al*. 1999). The SDM process entails the patient's and family's expression of their preferences and their discussion with the healthcare professional, who on his side elicits the patient's thoughts about pro and cons of the available treatments or options, aiming to reach agreement about healthcare decisions to be made (Elwyn *et al*. 2001). SDM is a component of a person‐centred care approach, a recognized theoretical framework that can guide the provision of high quality dementia care. Its aim was to acknowledge the identity and personhood of people with dementia. According to Edvardsson's review, the two key elements of a person‐centred care approach for people with severe Alzheimer disease are to take into account the person with dementia's point of view and to offer SDM (Edvardsson *et al*. 2008).

Reciprocity, by the contribution of the patient in the decision‐making process, is an important element that can improve health and well‐being in frail older people and that indirectly has an impact on the effectiveness of psychosocial interventions (Vernooij‐Dassen *et al*. 2001). Moreover, SDM seems to be the most typical pattern that occurs in decision‐making situations where the person with dementia, a family member and a professional caregiver are involved (Smebye *et al*. 2012). Despite this potential, SDM is not often used in LTC settings with persons with dementia or even with their family caregivers, whose views are frequently not included and documented in care planning (Cohen 1991, Broderick & Coffey 2013).

### Context

The study runs within the IMPACT project (Implementation of quality indicators in Palliative Care study) funded by the EU 7^th^ Framework Programme that involves five European countries, among which the Netherlands and Italy. Life‐and‐care plans, as tools for goal planning and for care and registration of treatment actions, are compulsory in both Dutch and Italian LTC facilities. In these settings, a multidisciplinary team assesses residents during the first two weeks following admission. When assessment is completed, a ‘life‐and‐care plan’ is developed and compulsorily signed by the professional responsible for the plan, the family caregiver and if possible the resident. In the Dutch LTC facility, usually a nurse is responsible for the plan and in the Italian setting, a nurse or any available and qualified member of the multidisciplinary team. The structure of the plans adopted in both countries is similar and consists of four main sections: Problems; Goals; Actions; Evaluation. Problem areas primarily cover: mental and physical well‐being; activities of daily living; and cognitive and social functioning. Plans are updated as changes in the resident's condition occur and at least once a year. The choice to develop and implement an SDM framework in care planning in the Netherlands and in Italy was primarily based on the existing collaboration between the University of Bologna and the University of Nijmegen. Second, SDM is an issue that is receiving growing attention in both countries. In the Netherlands, a policy called ‘family participation’ has been developed in the 1990s to promote the participation of family members in the care planning of their relatives admitted into nursing homes (Dijkstra 2007). However, a structured involvement of both family carers and dementia residents by using SDM in LTC settings has not become common practice. In Italy, the National Health Plan developed in 2011 underlines the importance of involving citizens and patients in the healthcare decision‐making process. However, there are only few studies on SDM carried out in this country and none of them was conducted in the dementia care area (Goss *et al*. 2011).

## Aim and objectives

This study has the following primary objectives: (1) to assess the feasibility and acceptability of an SDM framework in care planning to be used both to assess the preferences and (un)met needs of the LTC resident with dementia and his family caregiver and to plan tailored and shared actions based on the assessment outcomes; (2) to investigate how the process of SDM evolves between the resident, professional caregiver and family caregiver; (3) to investigate whether it is acceptable to professionals, residents and families becoming, embedded into the clinical practice of the involved LTC settings in Italy and the Netherlands.

The secondary objectives intend to explore the impact of the SDM framework on:

- the dementia residents' quality of life
- the family carers' quality of life and sense of competence
- the professional caregivers' job satisfaction
- the caregivers' satisfaction with the SDM intervention
- the organizational context where it takes place, i.e. barriers and facilitators, as perceived by the professionals.

In particular, we want to determine whether the SDM framework is likely to increase the number of residents' preferences and needs together with the actions taken to satisfy them recorded into their ‘life‐and‐care plans’ and whether it improves the residents' psychosocial well‐being.

## Design and methods

### Study design

The current project is a feasibility study. The research population consists of dementia residents living in the selected LTC settings, their main family carers and the professional caregivers usually taking care for the residents. The subjects are organized in triads: each triad is composed by the resident with dementia, the family and the professional caregiver. A multi‐method approach (Morse 2003) will be adopted to provide an in‐depth description of the SDM process developed in the triad. Quantitative data based on residents' personal files and on the screening and evaluation measures collected from professionals and family caregivers will be used.

### Subjects and settings

Two nursing home wards in the Netherlands and two nursing home wards in Italy are involved. In each country, one ward will randomly be assigned to the intervention group and the other to the control group. The same number of residents, family caregivers and healthcare professionals will be assessed in both groups and the same tools will be used. In the Netherlands two Dementia Special Care (DSC) units in the same nursing home will be recruited, whereas in Italy two different nursing homes will be enlisted, being similar in numbers of residents admitted, staffing patterns and level of medical and psychosocial care provided, as described in their charters of services. To avoid contamination, in the Netherlands the professionals working in the experimental DSC unit will not be the same as the ones working in the control DSC unit.

In each experimental and control nursing home ward, 20 dementia residents will be included, based on the following inclusion criteria: (1) having a diagnosis of dementia based on DSM IV (American Psychiatric Association 2000); (2) being able to give informed consent or, if legally incapable, having a family caregiver who can give informed consent for them; and (3) being supported by one primary family caregiver who agrees to participate and to be involved in the study too.

Inclusion criteria for the principal professional caregivers are: (1) being a member of the multidisciplinary team who is used to being directly involved in the care planning process; and (2) being a key staff member in the provision of residents' care and consequently to know the identified residents well. In both countries, at least eight healthcare professionals will be recruited to attend the training provided for the project and will later conduct the SDM interviews with 20 residents and their family caregivers.

Thus, the entire experimental group is composed by a total of 40 dementia residents, 40 family carers and about 16 healthcare professionals. These participants will be compared with the two other control nursing home wards that will be asked to involve the same number of subjects. After the selection is completed, a researcher will check the accuracy of the choices based on the requested inclusion criteria.

### Intervention

**Figure 1:**
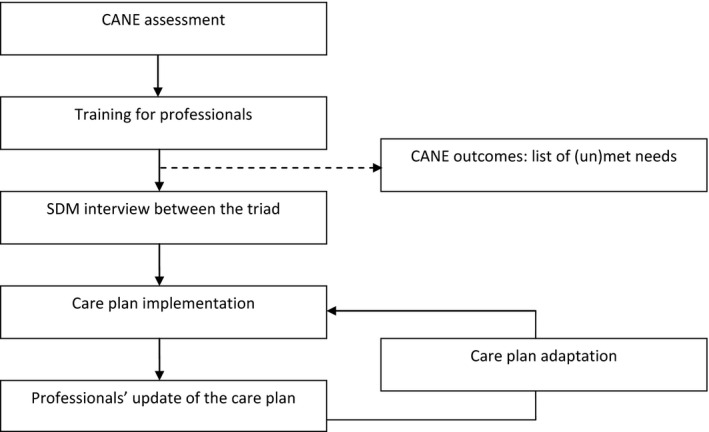
Graphical representation of the intervention,

As shown in Figure 1, the present project is a multifaceted intervention consisting of four phases to implement an SDM framework in (long‐term) care planning, to obtain a constantly developing plan that focuses not only on the medical, physical, psychosocial and spiritual needs of the residents, but that considers and documents their preferences and the actions taken by caregivers to meet them.

#### a) Pre‐intervention assessment − Dementia residents' (un)met needs assessment

At baseline (Table 1), a trained researcher will administer an adapted version of the Camberwell Assessment of Needs in the Elderly (CANE) (Orrell & Hancock 2004, Orrell *et al*. 2007) to the dementia residents and to the formal and informal caregivers. The CANE is a comprehensive, person‐centred needs assessment tool that has been designed for use with older people: the instrument is based on the principle that identifying a need means identifying a problem plus an appropriate intervention which will help or meet the need. It assesses the older person's needs from various perspectives: to reach this goal, CANE is to be administered not only to the older person but also to a key staff member and to an informal caregiver. The CANE has shown a good validity and reliability (Reynolds *et al*. 2000). In this study, only those items of the Dutch (Van der Roest *et al*. 2008) and Italian (Chattat & Celeste 2008) version of the CANE will be used to assess specific psychosocial needs of dementia persons who live in LTC settings. This was decided after discussion with the involved professionals as they declined the use of the full CANE questionnaire because of its length and relevance for nursing homes. They considered the need to manage behavioural problems, the need for tailored activities and the emotional and social needs as most important for residents with dementia in LTC settings (Cadieux *et al*. 2013). Starting from these data, we tried to improve the study protocol by discussing it with professionals to be involved. The items' relevance for nursing homes was related to a model for nursing home care, the Eden Alternative that aims to provide a person‐centered care environment for older residents (Brownie & Nancarrow 2013). On the basis of its principles, we have selected the CANE items that cover the following psychosocial issues: self‐care; daytime activities; psychological distress; information; behaviour; company and intimate relationship. The outcome of the selected CANE items will be a summary of met and unmet needs. The trained researcher who administers the CANE will share and discuss the information gained with the LTC professionals involved in the study before they will conduct the SDM interviews with the dementia resident and his family caregiver, so that they can use this information as a guidance for the interview. This will facilitate the selection and prioritization of their needs and the identification of possible interventions to meet them.

**Table 1:** Overview of outcomes measure

Variable	Instrument	Time of assessment	
		B	F1
***Baseline measurements***			
Demographic data of participants	Age, gender, educational status, marital status, employment	I	I
Descriptive data of LTC settings	Type of hierarchical organization, care models adopted, family carers' involvement, National Health System information, staff members' roles and education	I	I
***Patient***			
Needs assessment	Camberwell Assessment of Needs in the Elderly (CANE)	R/FC/C	R/FC/C
Level of dependency	Katz Activities of Daily Living index (ADL)	P/C	P/C
*Primary outcome measure*			
Documentation of residents' preferences and of the actions taken to satisfy them	Proportion of residents whose preferences and needs' satisfaction is documented	DR	DR
*Secondary outcome measure*			
Quality of life	Dementia quality of life Instrument (DQoL*)*	R	R
***Family caregivers***			
*Secondary outcome measure*			
Quality of life	EuroQOL	FC	FC
Sense of competence	Sense of Competence Questionnaire (SCQ)	FC	FC
***Professional caregivers***			
*Secondary outcome measure*			
Job satisfaction	Job Satisfaction Questionnaire	C	C
Assessment of the SDM professional attitude	Structured interviews	C	C
*Process measures*			
Assessment of the SDM interview process	Self‐report questionnaire	–	C/FC
Satisfaction with the SDM process; relevance, feasibility and maintenance of the intervention	Self‐report questionnaires	–	R/FC
Barriers/facilitators and influencing factors	Focus group interview	–	C/FC

B, baseline; F1, 6 months after the first SDM interview; P, patient file; C, professional caregiver as informant; I, structured interviews with participants; DR, documentation review; R, residents as informant; FC, family caregiver as informant.

#### b) Phase 1 – Training for professionals

Dementia experts with an expertise in teaching communication skills in the context of clinical care have developed a training for professionals, teaching them how to appropriately stimulate the residents with dementia during the SDM interview to facilitate the expression of their wishes and needs. This training will be provided to the professionals in the intervention wards of both LTC settings. The training programme will focus on the SDM principles in dementia care and active listening (Gordon 2000), to enhance the healthcare professionals' verbal and non‐verbal communication skills to be used to assess, meet and record the residents' needs and preferences during the SDM interview. Participants will receive a 2‐hour weekly training for 5 weeks. Each lesson will be guided by clearly defined learning goals and will be divided into three sessions: theory, role‐playing and feedback sessions.

- Theory sessions
  During these sessions, the healthcare professionals will learn the SDM model, active listening and self‐management principles as a guide to: (1) identify residents' problems or needs; (2) prioritize them, choosing the main needs or problems that will become the goals of the intervention; (3) identify alternatives to meet them; (4) decide and plan the intervention; (5) execute plans and (6) evaluate the outcomes.
- Role‐playing sessions
  During these sessions, professionals will practice skills and knowledge acquired in the theoretical part of the lesson. In some cases, the trainer will provide case‐vignettes that will be used as cues to set up role‐play exercises; in others professionals will be asked to report difficult situations they face during their daily work. Moreover, professionals will be invited to bring real care plans, to understand whether SDM is applied and to practice the learning objectives of the training programme in daily care situations.
- Feedback sessions
  The trainer will support and supervise the professionals during the role‐playing sessions, guide the discussion and provide feedbacks to stimulate reflection on their own professional attitude.

One additional lesson, 3 months after the end of the training, will be organized to discuss the problems professionals faced so far and to refresh some of the core issues of the training.

#### c) Phase 2 – SDM conversation

The SDM conversation will take place between a triad, composed of the resident with dementia, the family caregiver and the LTC professional as facilitator. The professional will be taught to tell the resident and the family caregiver that the aim of the consultation is to tailor the ‘life‐and‐care plan’ to the resident's actual needs and preferences. Using the unmet needs as collected with the CANE, as starting point, the main steps of the SDM process that will be applied during the conversation are: (a) identification of problems and needs; (b) prioritization of the most important problems or needs to set the intervention's goals; (c) discussion of options and preferences and (d) identification of actions. The role of the family caregiver is to support and facilitate the resident's expression: if communication is limited, the family caregiver is stimulated to intervene, to add information and to stimulate the person with dementia. Together, the participants in the consultation will make plans to comply with the prioritized needs and will develop actions to meet them.

#### d) Phase 3 – Implementation of plans

Immediately after the interview, the professional caregiver is asked to update the resident ‘life‐and‐care plan’ with the outcomes of the SDM interview reporting: I. the goals of the intervention based on the resident's problems and needs identified and preferences expressed, II. the planned actions based on the agreed decisions taken and III. the monitoring of the SDM intervention (i.e. the planned actions have been effectively implemented and/or the agreed decisions satisfied).

#### e) Phase 4 – Update

The ‘life‐and‐care plan’ is then updated regularly by the professional caregiver, who will report if all aspects of the intervention are (not) going according to plan.

## Measures

### Participants' details and LTC settings description

Demographics of the participants will be collected together with data on the inner organization and management of the involved LTC settings, considered potential influencing factors regarding the implementation process.

Besides, several valid instruments will be used. For a full description of the data collected and of the tools used (Table 1).

### residents' characteristics

#### Katz index of independence in Activities of Daily Living (ADL)

The Katz ADL (Katz *et al*. 1963) measures the clients' ability to independently perform activities of daily living. The Index ranks adequacy of performance in the six functions bathing, dressing, toileting, transferring, continence and feeding. Lower scores indicate a higher level of dependency. If the Katz index is not reported in the residents' medical record, the information will be gained by asking the units' key nurses or healthcare professionals to complete it. These data will be used as additional information to make a profile of the residents, to better identify and prioritize their main needs to be satisfied.

### Outcome measures

#### Primary outcome measure

The primary outcome measure is the proportion of dementia residents whose preferences, needs and related actions are known, satisfied and documented in their ‘life‐and‐care plan’ (Detering *et al*. 2010). Six months after the SDM interviews, a researcher will determine the compliance with residents' needs and wishes accomplished. The researcher will check the residents ‘life‐and‐care plan’ updated after the SDM interviews by professional caregivers, identifying any documentation of the resident' s needs and preferences, goal set by the triad, actions taken to satisfy it and goal satisfaction (see Phase 3 of the intervention).

#### Secondary outcome measures for the residents

##### Dementia quality of life Instrument (DQoL)

The DQoL is a reliable instrument to assess dementia patients' quality of life (Brod *et al*. 1999). It is administered in this study to measure the effects of applying the SDM framework on residents' quality of life. It is a 29‐items scale and one global item on overall quality of life. It directly assesses five domains of quality of life: positive affects, negative affects, feelings of belonging, self‐esteem and sense of aesthetics. Items are rated on 5‐point visual scales to facilitate the person with dementia' answers. In this study, given the impaired cognitive functioning of residents, the rating scale will be recoded and patients will answer yes or no to each question.

#### Secondary outcome measures for the family caregivers

##### EuroQOL

The EuroQOL (The EuroQol Group 1990) is used to assess family caregivers' quality of life. EuroQOL is a generic health‐related quality of life measure composed of five domains: mobility, self‐care, usual activities, pain/discomfort, anxiety/depression. It is valid and can be applied in the general population (Brazier *et al*. 1993).

##### Short Sense of Competence Questionnaire (SSCQ)

SSCQ (Vernooij‐Dassen *et al*. 1999) is used to assess the sense of competence of the family caregivers of dementia residents. It is a scale to be used for informal caregivers of older adults diagnosed with dementia. It consists of three domains: satisfaction with the demented person as a recipient of care, satisfaction with one's own performance and consequences of involvement in care for the personal life of the caregiver. It comprises seven items to be rated on a 5‐point scale (from very strongly agree to very strongly disagree). In this study, answers will be dichotomized (Vernooij‐Dassen 1993).

#### Secondary outcome measures for the professional caregivers

##### Job Satisfaction Questionnaire (JSQ)

The JSQ consists of 20 items, scored on a four‐point scale, from mostly negative to mostly positive and it consists of five factors: autonomy, competence, emotion, initiative and relation. High scores indicate high levels of job satisfaction (Orrung Walli *et al*. 2013). The factors have Cronbach's alpha coefficients between 0·74‐0·92 (Sellgren *et al*. 2008).

### Process measures

#### SDM interview process questionnaire

A questionnaire has been developed to measure how residents with dementia have been involved in the SDM process from the formal and informal caregivers' point of views. The questions have been developed by combining and adapting the items of two validated tools used to measure SDM in clinical encounters to be applicable in the nursing home situation (Kriston *et al*. 2010, Melbourne *et al*. 2010). Selection has been made based on the principles that will guide the SDM process with persons with dementia in LTC settings and that focus on: needs identification; options provision; advantages and disadvantages explanation; support to the clients in understanding the information given and in expressing their preferences and wishes; agreement about the final plans to satisfy them. Immediately after the SDM interviews, formal and informal caregivers will be asked to complete it.

#### Process evaluation measures

To explore caregivers' satisfaction with the SDM intervention, questionnaires with closed and open questions will be used. Moreover, data on the adherence rate (operationalized as the proportion of caregivers that actually adopt the intervention in the study), relevance, feasibility and maintenance of the intervention (operationalized as the extent to which the intervention is sustained over time) will be collected.

#### Focus group interviews

Focus group interviews with the involved professional caregivers will take place at the end of the project to collect suggestions, observations and opinions on barriers and facilitators to this practice in LTC settings, also considering and discussing national and setting‐related factors that could have affected the intervention's results, such as the organization of the National Health System, the national attention to the SDM attitude in healthcare settings or the nursing home's inner organization. Measurements related to LTC residents, professional and family caregivers will be performed at baseline and 6 months after the intervention (Table 1).

## Data analysis

Quantitative data will be analyzed using the Statistical Package for Social Science (SPSS). The proportion of residents whose SDM documentation on needs satisfaction has been fulfilled, will be analyzed using the Fischer's exact test. Secondary outcomes, at the level of residents, professional and informal caregivers, will be calculated using parametric and non‐parametric tests, making comparison between and within groups. Descriptive statistics will be used to compare the experimental and control nursing home wards for socio‐demographic characteristics and baseline variables. In each country, the focus group discussions' content will be translated into English. Two independent researchers will code the data by using a constant comparative method (Johnson *et al*. 2012). Each researcher will develop and label categories with appropriate codes outlining the core concepts of the focus group interviews. Then, the codes will be combined in clusters to define the concepts and identify similarities and differences between the interviews (Boeije 2002). Codings will be discussed until consensus will be reached.

## Ethical approval

In November 2013, the study has been approved by the ethics committee of both universities involved in the project in Italy and The Netherlands.

## Discussion

This article presents the protocol of a study to assess feasibility, acceptability and potential effectiveness of an SDM framework in care planning for long‐term care residents with dementia. The aim of the study was to explore whether it is effective and feasible to take the dementia residents' personal perspective into account regarding assessing and meeting their own needs through an SDM process with the professional and family caregivers. Thus, SDM is here considered an opportunity for persons with dementia to express their opinion and wishes and care planning a comprehensive and constantly developing process that should be based on the residents' preferences, not only on the professionals' or family carers' perspective.

### Strengths and limitations

The key element of this study is that it will contribute to our knowledge about the efficacy and of SDM interviews in nursing homes with persons with moderate to severe dementia and will consider the barriers and facilitators to this practice in LTC settings. Besides, it will make an important contribution to test the feasibility for a full trial, as recommended by the United Kingdom Medical Research Council guidance on the development and evaluation of complex intervention (Craig *et al*. 2008). Moreover, the study will take place in nursing homes located in two different countries, Italy and the Netherlands: these data represent a source of interesting information on the application and feasibility of this study in countries characterized by different cultures and healthcare systems organization.

At the same time, cultural differences may affect the primary and secondary outcomes: these data are therefore collected and considered during data analysis. Furthermore, only a few nursing homes are recruited in this study: their organization and residents population may not be representative of these parameters in both countries. In addition, the supportive presence of the family caregivers during the SDM interview may influence the resident's behaviour and attitude. Therefore, this co‐variable will be taken into account.

## Funding

The study has received funding from the European Union's Seventh Framework Programme FP7/2007‐2013 under grant agreement no. 258883.

## Conflict of interest

No conflict of interest has been declared by authors.

## Author contributions

All authors have agreed on the final version and meet at least one of the following criteria [recommended by the ICMJE (http://www.icmje.org/recommendations/)]:

- substantial contributions to conception and design, acquisition of data, or analysis and interpretation of data;
- drafting the article or revising it critically for important intellectual content.
